# On the increased prevalence of myasthenia gravis in patients with inflammatory bowel disease

**DOI:** 10.1055/s-0046-1817046

**Published:** 2026-03-23

**Authors:** Francisco de Assis Aquino Gondim, Marcellus Henrique Loiola Ponte de Souza, Lúcia Libanez Bessa Campelo Braga

**Affiliations:** 1 Universidade Federal do Ceará, Faculdade de Medicina, Departamento de Medicina Clínica, Serviço de Neurologia, Fortaleza CE, Brazil.; 2 Universidade Federal do Ceará, Faculdade de Medicina, Departamento de Medicina Clínica, Serviço de Gastroenterologia, Fortaleza CE, Brazil.

Dear Editor,

Dr. Christian Messina has written a Letter to the Editor 1 about our recent paper that depicted, for the first time in the literature, a statistically significant increase in the prevalence of myasthenia gravis (MG) and inflammatory bowel disease (IBD). 2 In this letter, Dr. Messina provided interesting comments about our findings that need to be clarified.

The first comment was about the possibility that our cases were pharmacologically-induced and not true autoimmune MG. None of our four MG patients with IBD was treated with any medication known to trigger MG, such as penicillamine or checkpoint inhibitors. One possible exception could be argued for patient 4, who was treated with infliximab. There is controversy in the literature about the possibility of MG induced by anti-tumor necrosis factor alpha (anti-TNFα) agents, especially etanercept. 3 Our patient had orbital apex syndrome due to posterior orbital pseudotumor followed by MG with anti-MUSK antibodies. To our knowledge, anti-MUSK positivity is not a feature of pharmacologically-induced MG. Patients 1 and 2 were never treated with infliximab. 4 Patient 3 had MG onset prior to her 1st treatment with infliximab. Since our paper included an extensive literature review and initially exceeded the word limit of the journal, we had to shorten several sections. Therefore, the information about the IBD drug treatments was not detailed.

The second point is that we did not conduct a prospective evaluation of all 606 IBD patients. This cohort study was started in 2004, and we do have a close collaboration between the Neurology and Gastroenterology Services. Thus, all clinically-relevant patients with either weakness or eye findings immediately undergo a neurological evaluation. 5 6 7 Nonetheless, we do agree that IBD patients treated with steroids or other immunosuppressive therapies could have their MG course masked. We have also raised this matter in a prior publication. 4

Regarding the diagnosis of small-fiber neuropathy (SFN), we do agree that the skin wrinkling test is not internationally recognized as a gold-standard test for the diagnosis of SFN, but most Brazilian institutions do not have access to skin biopsies with protein gene product 9.5 (PGP 9.5). In a previous Brazilian consensus about SFN, 8 we have listed possible alternatives for the diagnosis of SFN in Brazil, aware of the fact that not even skin biopsy with PGP 9.5 is exempt from false-positive results (the SFN findings could be mimicked by prior capsaicin treatment, for example). We agree that the issue of causality of SFN in IBD is still a matter of controversy, especially because IBD patients frequently present vitamin deficiencies, associated autoimmune conditions, and they are prone to the side effects of several medications. Despite that, it is striking to observe that all four MG patients also had sensory complaints at some point during their disease course, as well as markedly-abnormal skin-wrinkling test.

In summary, we would like to thank Dr. Messina for the important comments, and we agree that the MG prevalence can be higher; on the other hand, we were confident about the statistically significant association of increased MG prevalence in IBD patients. Further studies are necessary to confirm our findings and to further evaluate the neuropathy risks in IBD patients.
